# Evaluation as evolution: a Darwinian proposal for health policy and systems research

**DOI:** 10.1186/s12961-015-0007-x

**Published:** 2015-03-12

**Authors:** Alexandra L Martiniuk, Seye Abimbola, Merrick Zwarenstein

**Affiliations:** School of Public Health, University of Sydney, Australia and the George Institute for Global Health, University of Sydney, Sydney, 2006 NSW Australia; Dalla Lana School of Public Health, University of Toronto, Toronto, M5T 3 M7 ON Canada; National Primary Health Care Development Agency, Abuja, 900247 FCT Nigeria; Centre for Studies in Family Medicine, Department of Family Medicine, Schulich School of Medicine & Dentistry, Western University, London, N6A 3 K7 ON Canada; Institute for Clinical Evaluative Sciences, Toronto, M4N 3 M5 ON Canada

**Keywords:** Evaluation, Evolution, Health policy, Health systems

## Abstract

**Background:**

Health systems are complex and health policies are political. While grand policies are set by politicians, the detailed implementation strategies which influence the shape and impact of these policies are delegated to technical personnel. This is an underappreciated opportunity for optimising health systems. We propose that selective ‘breeding’ through successive evaluations of and selection among implementation strategies is a metaphor that health system thinkers can use to improve health care.

**Discussion:**

Similar to Darwinian evolution, the acceptance and accumulation of successful choices and the detection and discarding of unsuccessful ones would improve health systems in small and uncontroversial ways, over time. The effects of better implementation choices would be synergistic and cumulative, accumulating large impact (and lessons) from small changes. Just as with evolution of species, this means that even slight improvements over usual outcomes makes these numerous small choices as important a focus for system improvement as the overarching policy itself. Several alternative implementation approaches can be compared under real-world conditions in prospective head-to-head experimental and non-experimental explorations to understand whether and to what extent a strategy works and what works for whom, how, and under what circumstances in different locations. As in breeding or evolution, the best variants would spread to become the new, proven superior, implementation strategies for that policy in those settings.

**Conclusions:**

Evolution does not produce a new species whole, in a single transaction. Instead it gathers new parts and powers over time as different combinations are tested through competition with one another, to survive and spread or become extinct. Without necessarily changing or challenging grand policies, extending this idea to health systems innovation can facilitate thinking around how local, small – but cumulative – improvements in implementation potentially contribute to a pattern of successive adaptation spreading within its viable niche and ultimately providing locally-derived, long-term improvements in health systems.

## Background

Health systems, like the individuals and communities they serve, are complex and adaptive [1]. They include many sub-systems that interact with each other, and these interactions are affected by the individual or group behaviours of decision-makers, personnel, and users. Sheer complexity makes the impact of even the grandest of health policy decisions modest, unpredictable, and non-linear.

This complexity makes it very hard to know how a health system will respond to a specific policy, clinical intervention, or delivery method. The best method is to observe several sites responding to a given intervention, in comparison with other sites that respond differently to the intervention or those that did not receive the same intervention [1]. More than any other quantitative study design, a randomized trial can distinguish important intervention effects from chance variation and in an unbiased way and accurately answer the question: How much difference did a particular intervention make? [2]. Beyond this, qualitative research approaches can help us understand why an intervention works (or not), how, under what circumstances, for whom, and in which settings [3].

This commentary puts forward the suggestion that selective breeding is a metaphor that health care thinkers can use in their search for better implementation strategies (how one actions a policy) and health care delivery systems. The accumulation of successful choices and the death of unsuccessful ones would improve health systems over time. The metaphor of Darwinian evolution helps us think about the modification of innovations in different contexts and over time, as well as the accumulation of small innovations. The metaphor may also assist in understanding how an innovation came about, and why it was adopted and under what circumstances. This may help conceptually in health systems to think about health system innovation, adaptation, adoption, and modification based on context.

## Accumulating large lessons from small changes

Health policy is political [4]. Policymakers have goals which reflect the social values and interests they are promoting, influenced by public concerns and mass media attention to a problem, and, to a lesser extent, by data [5]. Other factors affecting their decisions may include path dependence, cost, corruption, level of technical capability, and public attitudes. Policies are rarely evaluated rigorously, reducing the possibilities for learning how to improve health systems.

Grand policies to address high level goals are publicly announced by the most senior elected officials to gain public and media attention, but the specific implementation plans are delegated to technical personnel working out of the public eye.

Constrained by resources, interests, and the inherited structure of their health systems, the number of distinct delivery options available to implement the new policy may be small. Because of their lower political sensitivity, comparative evaluation of these alternative implementation choices presents an underappreciated and potentially powerful opportunity for learning how to optimise health systems. Grand policies will remain politicized, but choices between alternative implementation strategies, being low key, are more amenable to scientific input. We are not suggesting an abandonment of efforts to improve policy. Nor are we making the argument that good implementation is more likely to improve health systems than policy. We are simply arguing that policies are often made by governments and influenced by other powerful actors, that the outcomes of policies alone are unpredictable, and that careful and objective selection of the most effective implementation strategies can result in the accumulation of small and beneficial changes.

Health system managers and frontline workers have to figure out an implementation strategy anyway. We argue this would best be done by formally comparing and testing alternative implementation strategies, giving the best chance of detecting those with superior outcomes. We suggest that these implementation choices be routinely compared, using head-to-head (direct comparison) quantitative and qualitative studies to identify the more effective implementation options under real-world conditions. In a complex system, the effects of better implementation choices would be synergistic and cumulative, varying from setting to setting and over time. Just as with evolution of species, this means that even slight improvements over usual outcomes makes these numerous small choices as important a focus for system improvement as policy itself. Of course, cumulative change is not always unproblematic and neither is it a simple process. Similarly, evaluation is not a panacea. Evaluation may demonstrate that none of the options is superior or optimal, and so fail to contribute to improvements in implementation, particularly as the context is constantly changing.

We recall a successful example in a resource-poor setting like South Africa, where nurse clinicians are the main providers of primary care and where in-service continuing professional development had largely been provided off site, interrupting clinical services and limiting sustainability and coverage. Two large pragmatic cluster randomised trials [6,7] with ambulatory primary care clinics as the unit of randomisation demonstrated clear impact improvements and acceptable cost effectiveness [8] due to a different in-service training approach (on-site, face-to-face team training by an outreach trainer), compared with usual off-site training. These evaluations resulted in acceptance and widespread dissemination of the particular implementation strategy that had been tested and, over time, this catalysed new attitudes to nurse-led primary care, a new national primary care guideline, and a new approach to building primary care capacity. Widespread national implementation [9] has so far trained over 12,000 nurses using this approach, and put over 1,000 outreach trainers in post, serving over half of all primary care clinics. Further successful testing [10], parallel qualitative evaluation [11], and widening of the scope of this training to cover chronic cardiovascular and mental health conditions is contributing to the spread of fully integrated primary care. This is now adapted [12] and under test [13] in a much lower income country. So, while national policy favouring nurse clinicians as providers had long been in place, a series of rigorous evaluations of a seemingly minor aspect of the policy, namely a test of a particular way of providing training for this cadre of health workers resulted in cumulative and far reaching impacts.

## What does nature tell us about iterative selection?

In nature, all forms of life spontaneously change. Nature repeatedly creates new variants of physiology or behaviour and selects the more successful through ‘survival of the fittest’ competition between these variants for scarce resources; this is evolution [14]. Over many iterations, species evolve: from microbes, to mammals, to humanity, each exquisitely adapted to a particular ecosystem, with a few species (since evolution is local) [15] spreading to occupy a global niche.

Humanity learned from evolution. The Darwinian process of preferential propagation of better variants is the founding principle of agriculture and animal husbandry. Selective breeding produces farm animals and crops. Over time, we chose their wild ancestors for domestication, and bred variants with large improvements in the traits that made their ancestors useful to us. These improvements in yield, pest, or drought resistance have been especially fast since formal agricultural experiments replaced intuitive selection.

We suggest that selective ‘breeding’ and successive ‘selection’ is a metaphor that health care thinkers can use in their search for better implementation strategies and health care delivery systems. Real-world head-to-head comparisons could help select the best implementation strategies. Collectively and cumulatively, consistent use of the successful choices would improve local health and economic outcomes, and some choices would spread widely.

## What would Darwinian health policy implementation look like?

Once a goal and a matching policy are defined to address a need, senior policymakers often delegate implementation to others. Those concerned with implementation may often be faced with several different but equally practical implementation strategies to reach the same policy goal. Their selection may be almost arbitrary, based on habit or tradition, with little or no evidence that any one is superior to another. Given that so little is at stake, there is the real possibility that, with appropriate support, they will be willing to test several alternative implementation approaches, which could then be compared rigorously under real-world conditions in several locations. These evaluations would identify the superior option (defined according to the policy goal, as well as the impact on health or health service outcomes such as effectiveness, efficiency, acceptability, or equity). The evaluations would also seek to understand how the different implementation strategies work (or not), for whom or in which settings, and under what circumstances [3].

As in breeding or evolution, the evaluation would reveal the best variants, which would spread to become the new, superior, way to implement that policy in those settings. Spread of successful implementation strategies to other, less similar settings might require further testing. Adaptation and selection are context specific [16-18]. Implementation strategies which are shown to be superior across a range of settings may have some underlying mechanism which can be understood, as a general principle for widespread use [19].

As mentioned above, it is not always the case that robust evaluation leads to ‘successful choices’. For instance, despite evidence dating back to the 1970s on the effectiveness of nurse practitioner services, contextual barriers have prevented their wide use in Canada [20]. Further, bad ideas or approaches do not always die off. Vestigial organs in evolution are one such example. Just as in natural selection in living beings there is never a perfect, successful organism but only one which best suits the environment at the time. As such, there are never ‘good’ or ‘bad’ ideas, or evolved functions. At one point in time, an organism (or a health service or program) may be ideal to its setting, but a feature of that setting may change suddenly – competitors may change, resources may disappear – rendering the organism/implementation strategy then poorly suited to its environment.

In evolution, as in health systems, the idea of how to measure ‘progress’ or ‘successful choice’ is skewed by the perspective of the observer. In nature, a plant’s perspective of a biologic feature may differ to a spider’s perspective of the same biologic feature. So, too, in health systems; for instance, removing patent rights to some pharmaceuticals may be seen as successful to some and a hindrance to others. As another example, an analysis of the movement from fee-for-service towards group practice and rostering (capitation) payment in Ontario family practice settings demonstrated that no single model can achieve the full range of policy objectives [21].

The strengthening of health care systems is a long-term process – there are no universal approaches that will rapidly improve the performance of every health system [22]. In biology, saltation – sudden change from one generation to the next – is rare. So, too, in health systems, the accumulation of multiple small advantages, each made apparent through evaluation, are more likely to get higher level support to be implemented more widely. A clinical example of cumulative change due to consistent rigorous testing would be the global reduction in morbidity and mortality from myocardial infarction as clinical care for this condition has improved, and these patterns of care have spread [23,24]. Indeed, the characteristics of successful health systems include not only learning from the improvement efforts of other health systems, but also the capacity to support and learn from local innovation by selecting promising strategies from a range of options through experimentation [22].

## Conclusions

Evolution does not produce a new species whole, in a single transaction. Instead, it gathers new parts and powers over time as different combinations are tested through competition with one another, to survive and spread or become extinct. Extending this idea to health systems innovation, better implementation choices could combine into wider delivery approaches. As each successive adaptation is tested to identify its contribution to the impact of care, and spreads accordingly, the scales tip towards cumulative long-term improvements in health systems.

Darwinian competitive selection generated multitudes of species in self-organising ecosystems and breeding raised the productivity of agriculture. Evaluations of many small implementation choices will incrementally evolve more effective and sustainable health care systems. Indeed, without necessarily changing grand policies, local, small – but cumulative – improvements in implementation can contribute to a pattern of successive adaptation spreading within its viable niche, ultimately providing locally derived, long-term improvements in health systems.

Ultimately, we are proposing the Darwinian metaphor to facilitate alternative ways of thinking that allows a greater emphasis on the importance of implementation strategies (not just policy), evaluation of implementation strategies, and incremental selection of advantageous strategies that, over time, cumulatively, and adapted to the local context, may assist in strengthening health systems. While Darwinian evolution occurs by natural selection, for iterative change in seemingly minor aspects (implementation) of a health system policy or innovation, we propose successive selection through randomised controlled experiments to identify the superior options, and mixed methods research and evaluation to reveal the mechanisms and contextual factors responsible for the outcomes of each implementation strategy.

## References

[1] Plsek PE, Greenhalgh T (2001). Complexity science: the challenge of complexity in health care. BMJ.

[2] Sackett DL, Wennberg JE (1997). Choosing the best research design for each question. BMJ.

[3] Pawson R, Tilley N (1997). Realistic evaluation.

[4] Morgan-Trimmer S (2014). Policy is political; our ideas about knowledge translation must be too. J Epidemiol Community Health.

[5] Innvaer S, Vist G, Trommald M, Oxman A (2002). Health policy-makers’ perceptions of their use of evidence: a systematic review. J Health Serv Res Policy.

[6] Fairall LR, Zwarenstein M, Bateman ED, Bachmann M, Lombard C, Majara BP (2005). Effect of educational outreach to nurses on tuberculosis case detection and primary care of respiratory illness: pragmatic cluster randomised controlled trial. BMJ.

[7] Zwarenstein M, Fairall LR, Lombard C, Mayers P, Bheekie A, English RG (2011). Outreach education for integration of HIV/AIDS care, antiretroviral treatment, and tuberculosis care in primary care clinics in South Africa: PALSA PLUS pragmatic cluster randomised trial. BMJ.

[8] Fairall L, Bachmann MO, Zwarenstein M, Bateman ED, Niessen LW, Lombard C (2010). Cost-effectiveness of educational outreach to primary care nurses to increase tuberculosis case detection and improve respiratory care: economic evaluation alongside a randomised trial. Trop Med Int Health.

[9] Colvin CJ, Fairall L, Lewin S, Georgeu D, Zwarenstein M, Bachmann MO (2010). Expanding access to ART in South Africa: the role of nurse-initiated treatment. S Afr Med J.

[10] Fairall L, Bachmann MO, Lombard C, Timmerman V, Uebel K, Zwarenstein M (2012). Task shifting of antiretroviral treatment from doctors to primary-care nurses in South Africa (STRETCH): a pragmatic, parallel, cluster-randomised trial. Lancet.

[11] Georgeu D, Colvin CJ, Lewin S, Fairall L, Bachmann MO, Uebel K (2012). Implementing nurse-initiated and managed antiretroviral treatment (NIMART) in South Africa: a qualitative process evaluation of the STRETCH trial. Implement Sci.

[12] Schull MJ, Cornick R, Thompson S, Faris G, Fairall L, Burciul B (2011). From PALSA PLUS to PALM PLUS: adapting and developing a South African guideline and training intervention to better integrate HIV/AIDS care with primary care in rural health centers in Malawi. Implement Sci.

[13] Sodhi S, Banda H, Kathyola D, Burciul B, Thompson S, Joshua M (2011). Evaluating a streamlined clinical tool and educational outreach intervention for health care workers in Malawi: the PALM PLUS case study. BMC Int Health Hum Rights.

[14] Darwin C (1999). The origin of species. Bantam Mass Market Edition.

[15] Rose MR, Passananti HB, Chippendale AK, Phelan JP, Matos M, Teotonio H (2005). The effects of evolution are local: evidence from experimental evolution in Drosophila. Integr Comp Biol.

[16] Sheikh K, Gilson L, Agyepong IA, Hanson K, Sssengooba F, Bennett S (2011). Building the field of health policy and systems research: framing the questions. PLoS Med.

[17] Bennett S, Agyepong IA, Sheikh K, Hanson K, Ssengooba F, Gilson L (2011). Building the field of health policy and systems research: an agenda for action. PLoS Med.

[18] Gilson L, Hanson K, Sheikh K, Agyepong IA, Ssengooba F, Bennett S (2011). Building the field of health policy and systems research: social science matters. PLoS Med.

[19] Bowen S, Zwi AB (2005). Pathways to “evidence-informed” policy and practice: a framework for action. PLoS Med.

[20] Edwards N, Rowan M, Marck P, Grinspun D (2011). Understanding whole systems change in health care: the case of nurse practitioners in Canada. Policy Polit Nurs Prac.

[21] Glazier RH, Klein-Geltink J, Kopp A, Sibley LM (2009). Capitation and enhanced fee-for-service models for primary care reform: a population-based evaluation. CMAJ.

[22] Mills A (2014). Health care systems in low- and middle-income countries. N Engl J Med.

[23] Briffa T, Hickling S, Knuiman M, Hobbs M, Hung J, Sanfilippo FM (2009). Long term survival after evidence based treatment of acute myocardial infarction and revascularisation: follow-up of population based Perth MONICA cohort, 1984–2005. BMJ.

[24] Yuan CT, Nembhard IM, Stern AF, Brush JE, Krumholz HM, Bradley EH (2010). Blueprint for the dissemination of evidence-based practices in health care. The Commonwealth Fund.

